# A new application of cell-free bone regeneration: immobilizing stem cells from human exfoliated deciduous teeth-conditioned medium onto titanium implants using atmospheric pressure plasma treatment

**DOI:** 10.1186/s13287-015-0114-1

**Published:** 2015-06-19

**Authors:** Masahiro Omori, Shuhei Tsuchiya, Kenji Hara, Kensuke Kuroda, Hideharu Hibi, Masazumi Okido, Minoru Ueda

**Affiliations:** Department of Oral and Maxillofacial Surgery, Nagoya University Graduate School of Medicine, 65 Tsurumai-cho, Showa-ku, Nagoya, 466-8550 Japan; EcoTopia Science Institute, Nagoya University, Furo-cho, Chikusa-ku, Nagoya, 464-8502 Japan

## Abstract

**Introduction:**

Surface modification of titanium (Ti) implants promotes bone formation and shortens the osseointegration period. The aim of this study was to promote bone regeneration and stability around implants using atmospheric pressure plasma (APP) pretreatment. This was followed by immobilization of stem cells from human exfoliated deciduous teeth-conditioned medium (SHED-CM) on the Ti implant surface.

**Methods:**

Ti samples (implants, discs, powder) were treated with APP for 30 seconds. Subsequently, these were immobilized on the treated Ti surface, soaked and agitated in phosphate-buffered saline or SHED-CM for 24 hours at 37 °C. The surface topography of the Ti implants was observed using scanning electron microscopy with energy dispersive X-ray spectroscopy. In vivo experiments using Ti implants placed on canine femur bone were then conducted to permit histological analysis at the bone-implant boundary. For the in vitro experiments, protein assays (SDS-PAGE, Bradford assay, liquid chromatography-ion trap mass spectrometry) and canine bone marrow stromal cell (cBMSC) attachment assays were performed using Ti discs or powder.

**Results:**

In the in vitro study, treatment of Ti implant surfaces with SHED-CM led to calcium phosphate and extracellular matrix protein immobilization. APP pretreatment increased the amount of SHED-CM immobilized on Ti powder, and contributed to increased cBMSC attachment on Ti discs. In the in vivo study, histological analysis revealed that the Ti implants treated with APP and SHED-CM stimulated new bone formation around implants.

**Conclusions:**

Implant device APP pretreatment followed by SHED-CM immobilization may be an effective application to facilitate bone regeneration around dental implants.

## Introduction

Titanium (Ti) implants are widely used for the restoration of missing teeth. However, Ti by itself does little to promote new bone formation on the surface of the Ti implant. This bone formation process, known as osseointegration, delays implant loading and tends to increase implant survival time. Moreover, bone-implant contact (BIC) is the percentage of the implant surface in contact with bone. A high BIC value indicates greater implant stability. However, there are a number of problems with current implantation methods. First, it takes several months to obtain sufficient implant stability. Second, bone morphogenesis is often limited around the Ti implant [1]. New biomaterials are therefore required to shorten the osseointegration period and promote BIC [2, 3].

Studies have shown that osseointegration can be modulated by implant surface properties [4]; for example, rough surfaces promote osseointegration more effectively than machined surfaces [5]. A number of treatments are used to modify implant surface properties. Mechanical and chemical treatments such as sand blasting and acid etching [6], anodization [7, 8], or hydroxyapatite coating [9, 10] are used to modify the surfaces of Ti implants [11], promoting osteogenesis and thus early osseointegration. In addition to these mechanical and chemical treatments, hydrophilic treatments such as atmospheric pressure plasma (APP) treatment [12–14], UV treatment [15] and hydrothermal treatment [16] have also been used to obtain early osseointegration. The effect of these hydrophilic treatments is protein immobilization promotion as a result of hydrocarbon removal from the Ti surface [17].

Researchers have even recently attempted to engraft bone marrow stromal cells (BMSCs) or umbilical cord stem cells onto the implant surface to improve osseointegration [18, 19]. The methods used for cell engraftment, however, were complicated, and resulted in poor cell differentiation and survival rates [20]. Biological molecules such as BMP-2 [21], type I collagen [22], fibronectin [23], amelogenin [24], and an RGD peptide [25] were then added along with the stem cell implant to try and better simulate the microenvironment of bone and promote osseointegration [26].

We have previously attempted to build on these surface modification approaches by immobilizing mesenchymal stem cell-conditioned medium (MSC-CM) on the implant surface. Conditioned medium (CM) is a potentially useful tool for stimulating bone regeneration because cultured MSCs secrete various growth factors and cytokines into the medium that have the capability of stimulating tissue regeneration [27, 28]. CM offers a convenient method to promote tissue regeneration/healing because it is easy to obtain large quantities of this medium with uniform quality [29–31]. We previously reported that immobilization of CM derived from BMSCs on Ti implants promoted osteogenesis around the implant, and contributed to early stability after implantation [32]. CM derived from BMSCs contains cytokines, growth factors, and extracellular matrix (ECM) components [33] that play important roles in the regeneration of bone around the Ti implants. Recently, stem cells from human exfoliated deciduous teeth (SHED) were used for bone regeneration [34]. SHED are a population of highly proliferative postnatal stem cells capable of differentiating into odontoblasts, adipocytes, neural cells, and osteogenic cells [35]. Additionally, SHED have a higher capacity to undergo differentiation than bone marrow-derived mesenchymal stem cells [36, 37]. We hypothesized that APP pretreatment followed by immobilization of SHED-conditioned medium (SHED-CM) on the surface of the Ti implant may promote osteogenesis around the implant, thus facilitating early osseointegration. To investigate this hypothesis, SHED-CM from cultured exfoliated deciduous teeth-derived cells was immobilized on a commercially available Ti implant pretreated with APP. The components of biomolecules in the SHED-CM immobilized on the surface of pretreated Ti implants, the initial attachment of canine bone marrow stromal cells (cBMSCs) to the Ti discs, and the stability of the Ti implants after implantation into dog femurs were analyzed. We demonstrate that APP pretreatment increases the amount of SHED-CM-derived proteins immobilized on the implant surface, and promotes the attachment of cBMSCs onto the Ti surface, thereby contributing to early osseointegration.

## Methods

### Ti materials

A Brånemark MK III TiUnite® threaded external hex (diameter 3.75 mm, length 7 mm; Nobel Biocare, Gothenberg, Sweden) was used in our experiments. Pure grade I Ti discs (diameter 15 mm) were purchased from Ofa Co. Ltd (Chiba, Japan). Ti powder (1–2 mm particle size) was obtained from Rare Metallic Co. Ltd (Tokyo, Japan).

### Cell culture

Human dental pulp tissues were obtained from clinically healthy, extracted deciduous teeth from patients aged 6–12 years old. The consents were obtained from all patients to establish SHED samples. The Nagoya University Ethics Committee approved the experimental protocols from ethical and scientific points of view. The SHED were a gift from Kiyoshi Sakai [38]. cBMSCs were isolated from the aspirated iliac bone marrow of hybrid dogs (18–36 months old, weight 15–25 kg). The single cell suspension of dental pulp/canine iliac bone marrow was seeded onto culture dishes. The dishes were then cultured at 37 °C and 5 % CO_2_ in Dulbecco’s modified Eagle’s medium (DMEM; Sigma-Aldrich, St. Louis, MO, USA), and supplemented with 10 % fetal bovine serum (FBS; Gibco, Rockville, MD, USA). Further, 1 % antibiotic-antimycotic (100 units/mL penicillin G, 100 mg/mL streptomycin, and 0.25 mg/mL amphotericin B; Gibco) was added to the culture. After 3 days of culture, floating cells were removed and the medium was replaced with fresh medium. Subsequently, the medium was changed once every 2 days. Spindle-shaped cells that adhered onto the plastic dish were passaged when the cells approached confluence using 0.05 % trypsin-EDTA (Gibco). The cells belonging to passages 3–9 were used for experiments as the SHED or cBMSCs.

### Preparation of SHED-CM

SHED-CM was prepared in accordance with published methods [39]. The cell culture medium was changed to serum-free DMEM after SHED reached 70–80 % confluence. After 48 hours incubation at 37 °C and 5 % CO_2_, the culture medium was collected and centrifuged at 22,140 × *g* for 5 minutes at 4 °C. After brief re-centrifugation at 44,280 × *g* for 3 minutes at 4 °C, the supernatant was collected and was used as SHED-CM.

### APP treatment on Ti materials

The implants were treated with APP using a system power of 400 W with N_2_ gas (MPS-01K01C, Kurita factory Co. Ltd Japan). The distance between the plasma pen (the end of the discharge capillary) and implant was set at 5 mm. The length of the free-burning plasma plume was 10 mm, and the plasma treatment time was 30 s. Ti powder and Ti discs were treated using the same method.

### Immobilization of SHED-CM on Ti materials

Immediately after APP treatment, Ti samples were soaked and agitated in SHED-CM for 24 hours at 37 °C. As a control, plasma-treated and plasma-untreated Ti samples were soaked and agitated in phosphate-buffered saline (PBS). After treatment, the Ti materials were washed three times with 10 mL PBS. Samples were then divided into four groups: the plasma-untreated Ti on which PBS was immobilized (N-PBS), plasma-treated Ti on which PBS was immobilized (P-PBS), plasma-untreated Ti on which SHED-CM was immobilized (N-CM), and plasma-treated Ti on which SHED-CM was immobilized (P-CM).

### Characterization of the Ti implant surface

Implant samples were prepared according to standard procedure. Briefly, a 30-μm thick osmium coating was applied to the surface of Ti materials with an osmium plasma coater (NL-OPC80NS; Japan laser electron Co. Ltd, Tokyo, Japan). The surface topography of the Ti implant was examined by scanning electron microscopy (SEM) (S-800S; Hitachi High-Technology, Tokyo, Japan) alone, and SEM (S-4800; Hitachi High-Technology) combined with energy dispersive X-ray spectroscopy (SEM-EDX) (HORIBA-EMAX80; Hitachi High-Technology). Imaging was performed at 10 kV and 3.3 A. N-CM implants were treated with 4 M guanidine (Sigma-Aldrich) or 10 % EDTA (Sigma-Aldrich) for 15 minutes at 37 °C. These N-CM implants were then observed using SEM-EDX with identical parameter settings to CM implants.

### Protein assay

Proteins in SHED-CM immobilized on 30 g Ti powder were extracted with distilled water, 4 M guanidine, or 10 % EDTA. The extracts were dialyzed for 5 days in a Visking tube (Nihon Medical Science Co. Ltd, Osaka, Japan) against 7.5 L distilled water. The samples were frozen and then dried using a FreeZone Freeze Dry System (FZ-1; LABCONCO, Kansas, MO, USA). Proteins extracted using 4 M guanidine were quantified using a Bradford protein assay [40] and then analyzed by 10 % SDS-PAGE. This protein analysis was then followed by silver staining (Pierce® Color Silver Stain Kit; Thermo Scientific, Rockford, IL, USA) and Coomassie Brilliant Blue (CBB) staining according to standard procedure. The extracted proteins and an undiluted solution of SHED-CM were analyzed using liquid chromatography-ion trap mass spectrometry (LC/MS/MS). In-solution protein digestion was carried out through alkylation, demineralization, and concentration steps in order to prepare proteins for LC/MS/MS analysis. In the alkylation step, samples were mixed with 7 M guanidine hydrochloride (WAKO Pure Chemical Industries, Osaka, Japan) in distilled water. To this solution, 20 μL of 3 M Tris-HCl (pH 8.5) and 10 μL of 0.1 M DTT (WAKO Pure Chemical Industries) were added. The mixture was then allowed to stand for 30 minutes at room temperature. Following this, 10 μL of 0.2 M iodoacetamide (WAKO Pure Chemical Industries) was added, and the mixture was incubated for 1 hour at room temperature in the dark. In the demineralization and concentration steps, chloroform methanol precipitation was performed. In the last step, a trypsin digest was performed for 16 hours at 37 °C with 10 μL urea, 40 μL of 0.1 M Tris-HCl (pH 8.5), and 0.5 μL of 1 μg/μL Trypsin Gold for MS (Promega KK, Tokyo, Japan) diluted in 50 mM acetic acid (WAKO Pure Chemical Industries). After digestion, samples were centrifuged at 20,000 × *g* for 20 minutes at 4 °C, and the middle layer containing proteins in three layers was collected. Nanoelectrospray tandem mass spectrometric analysis was then performed using an LCQ Advantage mass spectrometry system (Thermo Finnigan, Waltham, MA, USA) in series with a Paradigm MS4 HPLC System (Michrom BioResources, Auburn, CA, USA). Samples were injected onto the Paradigm MS4 HPLC System equipped with a Magic C18AQ column (diameter 0.1 mm, length 50 mm; Michrom BioResources). Reverse-phase chromatography was performed by applying a linear gradient (0 minutes, 95 % A and 5 % B; 45 minutes, 0% A and 100 % B) of solvent A (2 % acetonitrile with 0.1 % formic acid) and solvent B (90 % acetonitrile with 0.1 % formic acid) at a flow rate of 1 μL/minute. Ionization for mass spectrometry was performed using an ADVANCE Captive Spray Source (Michrom BioResources) at a capillary voltage of 1.6 kV and a temperature of 150 °C. Prior to MS/MS analysis, a precursor ion scan was carried out using a 400 to 2,000 mass to charge ratio (m/z). Multiple MS/MS spectra were submitted to the Mascot program, version 2.4.1 (Matrix Science, Boston, MA, USA) for the MS/MS ion search.

### Cell attachment assay

The cBMSCs (1.0 × 10^5^) were seeded on treated Ti discs (n = 3) and cultured at 37 °C and 5 % CO_2_ for 1 or 24 hours. The cBMSCs that adhered to Ti discs were removed using incubation with 0.05 % Trypsin-EDTA (Gibco) for 5 minutes. Ti discs were examined to ensure that no cBMSCs remained attached. The detached cells were counted with the help of a hemocytometer (Sunlead Glass, Tokyo, Japan). After 24 hours, cultured cBMSCs were fixed with 4 % paraformaldehyde (Sigma-Aldrich) and stained with desired fluorescent dyes followed by 100 nM DAPI (Roche Applied Science, Basel, Switzerland), and 100 nM rhodamine phalloidin (Cytoskeleton, Inc., Denver, CO, USA) stains. After histological staining, the cells were visualized using a confocal laser-scanning microscope (A1+; Nikon, Tokyo, Japan).

### In vivo experiments

All animal experiments were reviewed and approved in advance by the ethics committee of the Nagoya University School of Medicine. Surgical procedures were performed as reported previously [41]. Briefly, hybrid dogs (aged 18–36 months, weight 15–25 kg) were operated under general anesthesia induced by intravenous administration of pentobarbital (Somnopentyl®; Kyoritsu Seiyaku, Tokyo, Japan) used at 20 mg/kg body weight. Following hair shaving and cleaning with iodine solution at the femur and surgical surrounding area, a 5-cm incision was made at the skin level. The flap was reflected and the radius diaphysis was exposed. The initial drilling was performed using a 2-mm diameter pilot drill at 2,000 rpm. Then, low-speed sequential drilling with burs of 2.4, 2.8, and 3.2 mm was performed at 2,000 rpm, and the osteotomy sites were unicortical defects. The procedure included irrigation with cold saline during drilling to reduce the heat from friction. A total of 32 implants (n = 4) was inserted into femurs, 1.5 cm apart, using a dental implant device (Implanter Neo; Kyocera Medical, Osaka, Japan). The surgical wound was then closed carefully with 4-0 absorbable surgical suture (Atom vet’s medical, Kyoto, Japan). Post-surgical management involved intake of antibiotics (Azithromycin; Pfizer, Tokyo, Japan) daily for 3 days, a soft diet, and topical application of 2 % chlorhexidine (Dainippon Sumitomo Pharma, Osaka, Japan) twice a week. At 4 or 8 weeks after the implantation, the dogs were given general anesthesia and euthanized by exsanguination following the administration of heparin sodium (400 U/kg) and were perfused with 10 % formalin (WAKO Pure Chemical Industries).

### Radiological and histological analysis

Samples were visualized using a laboratory micro-CT machine (R_mCT2; Rigaku Co., Tokyo, Japan). Three-dimensional image-analysis software (TRI/3D-BON; Ratoc System Engineering, Tokyo, Japan) was then used to construct three-dimensional images of these samples. Samples were then embedded in Technovit 7200® (Okenshoji Co. Ltd, Tokyo, Japan) for histological analysis. Each block was cut along the long axis of the implant into 30-μm thick sections. The sections were stained using 0.05 % toluidine Blue (Muto Chemical Co. Ltd, Tokyo, Japan) according to standard methods. The BIC and bone area fraction occupancy (BAFO) were analyzed with published methods [12]. Digital images of sections were analyzed with image-analysis software (VMS-50 VideoPro®, Inotech Corporation, Hiroshima, Japan) after computer-based histomorphometric measurements. BIC (%) was estimated using the following equation BIC (%) = direct implant bone contact/peri-implant length. The BAFO between plateaus was determined with the help of Image J (Ver.1.46 K; [42]) from confocal microscopy images. The percentage area occupied by bone was calculated from the total area within the implant thread.

### Statistical analysis

Statistical differences were evaluated with the help of Tukey’s HSD (honestly significant difference) test (IBM SPSS statistics 21, Armonk, NY, USA). Digitized quantitative of SHED-CM, counts of attached cells, BIC, and BAFO have been expressed as means ± standard deviations. The threshold for statistical significance was set at *P* < 0.05.

## Results

### Topographical characterization of the Ti implant surface treated with APP and SHED-CM

**Fig. 1:**
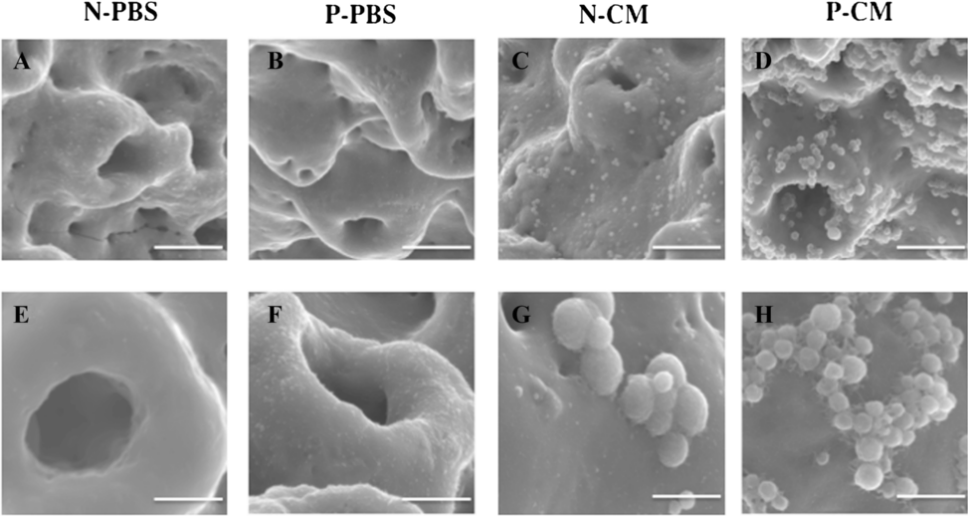
Topology of Ti implant surface, analyzed using SEM. Under × 10,000 magnification (**a**-**d**); under × 30,000 magnification (**e**-**h**). (**a**, **e**) Plasma-untreated Ti on which PBS was immobilized (N-PBS) implants, (**b**, **f**) plasma-treated Ti on which PBS was immobilized (P-PBS) implants, (**c**, **g**) plasma-untreated Ti on which SHED-CM was immobilized (N-CM) implants, (**d**, **h**) plasma-treated Ti on which SHED-CM was immobilized (P-CM) implants. Bars indicate 3 μm (**a**–**d**) and 1 μm (**e**–**h**)

**Fig. 2:**
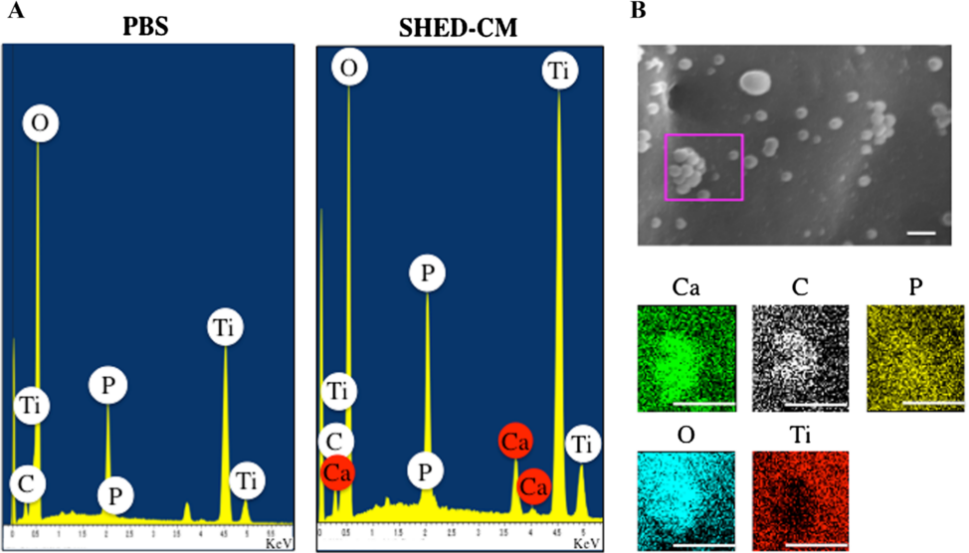
Characterization of Ti implant surface using SEM-EDX. SEM-EDX spectrum of the phosphate-buffered saline (PBS) group and stem cells from human exfoliated deciduous teeth-conditioned medium (SHED-CM) group (**a**), and SEM images and X-ray mapping of elemental calcium (Ca), carbon (C), oxide (O), phosphate (P), and titanium (Ti) of P-CM in the pink frame (**b**). Bar indicates 1 μm

The SEM images revealed differences between the treatments on the Ti implant surface. Under × 10,000 magnification, the surface of N-PBS and P-PBS showed only projections of TiUnite®, with roughness of several micrometer in thickness. In contrast, the N-CM and P-CM surfaces showed attached round-shaped deposits (Fig. 1c, d, g, h) that were uniformly distributed on the Ti implants. Further, a greater abundance of attached deposits in P-CM than in N-CM was observed. Under × 30,000 magnification, the deposits had an aggregated appearance. Deposit diameter was approximately 350 nm. Additionally, needle-shaped structures were visible at the interface of the substrate and implant. SEM-EDX spectra of N-CM and P-CM revealed the presence of calcium (Ca), carbon (C), phosphate (P), oxide (O) and Ti, whereas those of N-PBS and P-PBS showed the presence of only C, P, O and Ti (Fig. 2a). X-ray mapping of SEM images revealed high concentrations of Ca, C, P and O, and a low abundance of Ti in the round-shaped deposits in the CM implants (Fig. 2b).

### Quantification and identification of proteins derived from SHED-CM on the Ti surface

**Fig. 3:**
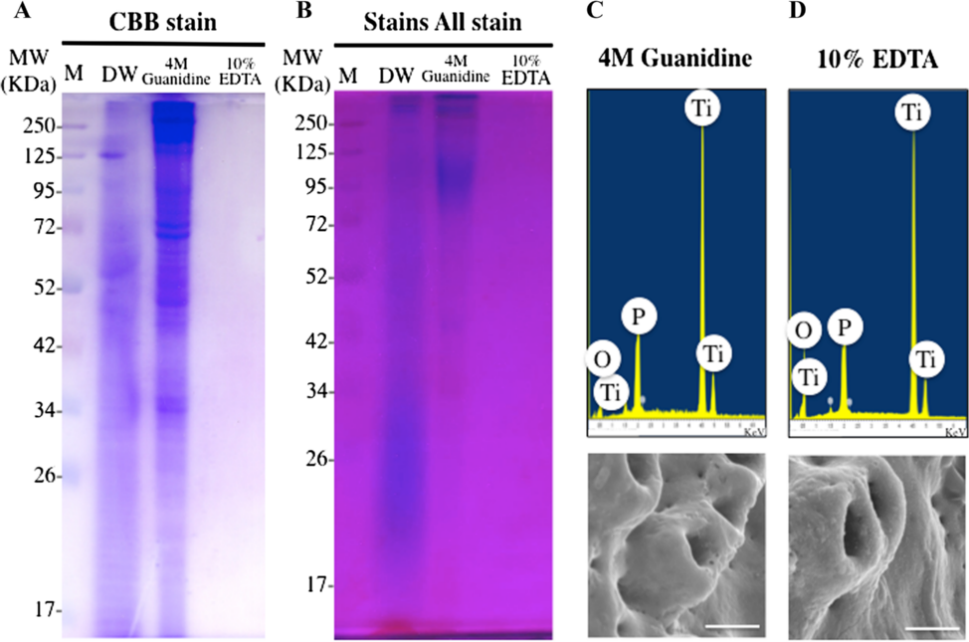
Protein extraction of Ti surface and relationship between protein and Ca components. Coomassie Brilliant Blue (CBB) (**a**) and Stains All (**b**) were used to stain the SDS-PAGE gel after loading the extracts of protein immobilized on titanium powder. Distilled water (DW), 4 M guanidine, or 10 % EDTA was used for extraction. SEM images and SEM-EDX spectrum of 10 % EDTA (**c**) and 4 M guanidine (**d**). Bar indicates 1 μm. *MW* Molecular weight, *M* Marker, *O* oxide, *P* phosphate, *Ti* titanium

**Fig. 4:**
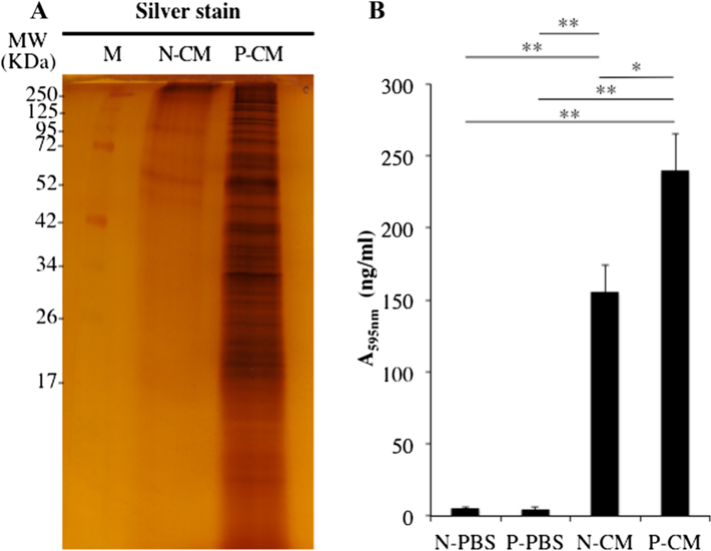
Quantification of protein derived from SHED-CM by using APP pretreatment. Silver staining of proteins separated by SDS-PAGE. Proteins immobilized on titanium powder (N-CM and P-CM) were extracted using 4 M guanidine (**a**). Results of the Bradford protein assay (**b**). Data from panel (**b**) are presented as mean ± SD (n = 3). *P < 0.05, **P < 0.01. *M* Marker, *N-CM* plasma-untreated Ti on which SHED-CM was immobilized, *N-PBS* plasma-untreated Ti on which PBS was immobilized, *P-CM* Plasma-treated Ti on which SHED-CM was immobilized, *P-PBS* Plasma-treated Ti on which PBS was immobilized

Results of SDS-PAGE analysis showed the presence of protein in PBS and guanidine extracts from Ti powder. In contrast, EDTA extracts did not contain any detectable amounts of protein (Fig. 3a, b). Guanidine is known to denature proteins and was used to extract the proteins immobilized onto the Ti powder. EDTA is a chelating reagent that removes Ca^2+^ and Mg^2+^ ions and was used to extract the Ca^2+^ component of the Ti powder. After treatment with guanidine and EDTA, a SEM-EDX spectrum of P-CM implant surfaces showed that Ca was absent on their Ti surfaces. SEM images confirmed a lack of attached substances (Fig. 3c, d). The results of silver staining of SDS-PAGE gels showed immobilized proteins on Ti powder and revealed higher amounts of protein in P-CM than in N-CM (Fig. 4a). Results of the Bradford protein assay showed that the quantity of protein immobilized on the Ti surface was significantly higher in P-CM than in N-CM (Fig. 4b). LC/MS/MS analysis identified the presence of various proteins in SHED-CM (Table 1) and in the 4 M guanidine extracts of treated Ti powder (Table 2). However, these proteins were not found in the 10 % EDTA extracts (Table 3). The proteins that attached to Ti primarily consisted of ECM proteins such as collagen type I, fibronectin, and decorin.

**Table 1:** Proteins identified in stem cells from human exfoliated deciduous teeth-conditioned medium

Protein name	Accession No.	Sequence
Collagen alpha-2 (I) chain	P02464	R.GAPGAVGAPGPAGATGDR.G
Collagen alpha-2 (I) chain	P02452	K.STGGISVPGPMGPSGPR.G
Vimentin	P08670	R.QDVDNASLAR.L
Collagen alpha-1 (IV) chain	P12109	R.GAPGPAGPPGDPGLMGER.G
IGF-binding protein 7	Q16270	K.HEVTGWVLVSPLSK.E
Fibronectin	P02751	K.VTIMWTPPESAVTGYR.V
Decorin	P07585	K.DLPPDTTLLDLQNNK.I
Plasminogen activator inhibitor 1	P05121	R.QFQADFTSLSDQEPLHVAQALQK.V
Actin, cytoplasmic 2	P60709	K.SYELPDGQVITIGNER.F
Sulthydryl oxidase 1	O00391	R.LAGAPSEDPQFPK.V
SPARC	P09486	R.LEAGDHPVELLAR.D
Metalloproteinase inhibitor 1	P01033	K.GFQALGDAADIR.F
Collagen alpha-2 (IV) chain	P08123	K.GAPGLAGKNGTDGQK.G

**Table 2:** Protein identified in stem cells from human exfoliated deciduous teeth-conditioned medium immobilized to titanium; extraction using 4 M Guanidine

Protein name	Accession No.	Sequence
Collagen alpha-2 (I) chain	P02464	R.GEAGAAGPAGPAGPR.G
Fibronectin	P02751	R.ESKPLTAQQTTK.L
Collagen alpha-2 (I) chain	P02452	K.GLTGSPGSPGPDGK.T
Vimentin	P08670	K.ILLAELEQLK.G
Decorin	P07585	K.ILLAELEQLK.G
IGF-binding protein 7	Q16270	K.ITVVDALHEIPVK.K
Follistatin-rerated protein 1	Q12841	R.YVQELQK.H
Metalloproteinase inhibitor 1	P01033	K.GFQALGDAADIR.F

**Table 3:** Protein identified in stem cells from human exfoliated deciduous teeth-conditioned medium immobilized to titanium; extraction using 10 % EDTA

Protein name	Accession No.	Sequence
ND	ND	ND

*ND* Not Detected

### Effect of APP and SHED-CM on the cBMSC attachment to the Ti surface

**Fig. 5:**
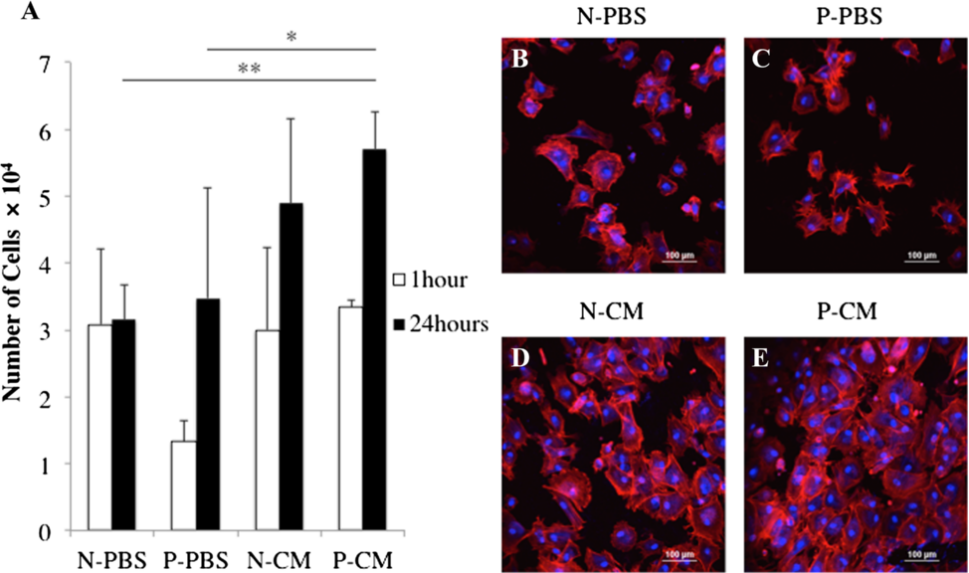
Effect of APP and SHED-CM on cBMSC attachment to the Ti surface. The detached cBMSCs were counted with the help of a hemocytometer (**a**). Data are presented as mean ± SD (n = 3). *P < 0.05, **P < 0.01. Confocal microscopy images of cBMSCs 24 hours after seeding (**b**-**e**). DAPI for nuclei (blue) and rhodamine phalloidin for actin filaments (red). *N-CM* plasma-untreated Ti on which SHED-CM was immobilized, *N-PBS* plasma-untreated Ti on which PBS was immobilized, *P-CM* Plasma-treated Ti on which SHED-CM was immobilized, *P-PBS* Plasma-treated Ti on which PBS was immobilized

The number of cells attached to the Ti discs was not significantly different among the four experimental groups after 1 hour in culture. However, after 24 hours in culture, the number of attached cells was significantly higher in the P-CM groups than either the N-PBS or P-PBS groups (Fig. 5a). Phalloidin and DAPI staining further verified this observation by showing that the number of attached cells was higher in N-CM and P-CM groups than in N-PBS and P-PBS (Fig. 5b–e). The morphology of cBMSCs was similar in all experimental groups.

### Micro-CT analysis of implant treated with APP and SHED-CM

**Fig. 6:**
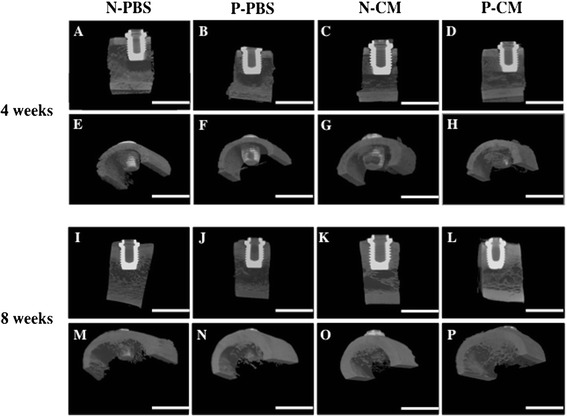
Micro-CT images of Ti implants inserted into the canine’s femur bone in vivo*.* X-ray images of bone formation around the Ti implants 4 weeks after implantation (**a**–**h**) and 8 weeks after implantation (**i**–**p**). (**a**, **e**, **i**, **m**) N-PBS implants; (**b**, **f**, **j**, **n**) P-PBS implants; (**c**, **g**, **k**, **o**) N-CM implants; (**d**, **h**, **l**, **p**) P-CM implants. Bar indicates 5,000 μm. *N-CM* plasma-untreated Ti on which SHED-CM was immobilized, *N-PBS* plasma-untreated Ti on which PBS was immobilized, *P-CM* Plasma-treated Ti on which SHED-CM was immobilized, *P-PBS* Plasma-treated Ti on which PBS was immobilized

Calcification around the implant fixture was evaluated using micro-CT. Images collected 4 and 8 weeks after implantation showed that radiopacities indicating calcified tissue formation were greater in N-CM and P-CM than in N-PBS (Fig. 6). Further, the opacity was significantly higher in P-CM than the other groups. In the P-CM groups, the radiopacities were observed not only around the implant interface, but also at the sides away from implants. In contrast, the bottom of the implant showed no significant radiopacities in all groups.

### Bone morphogenesis around the Ti implant

**Fig. 7:**
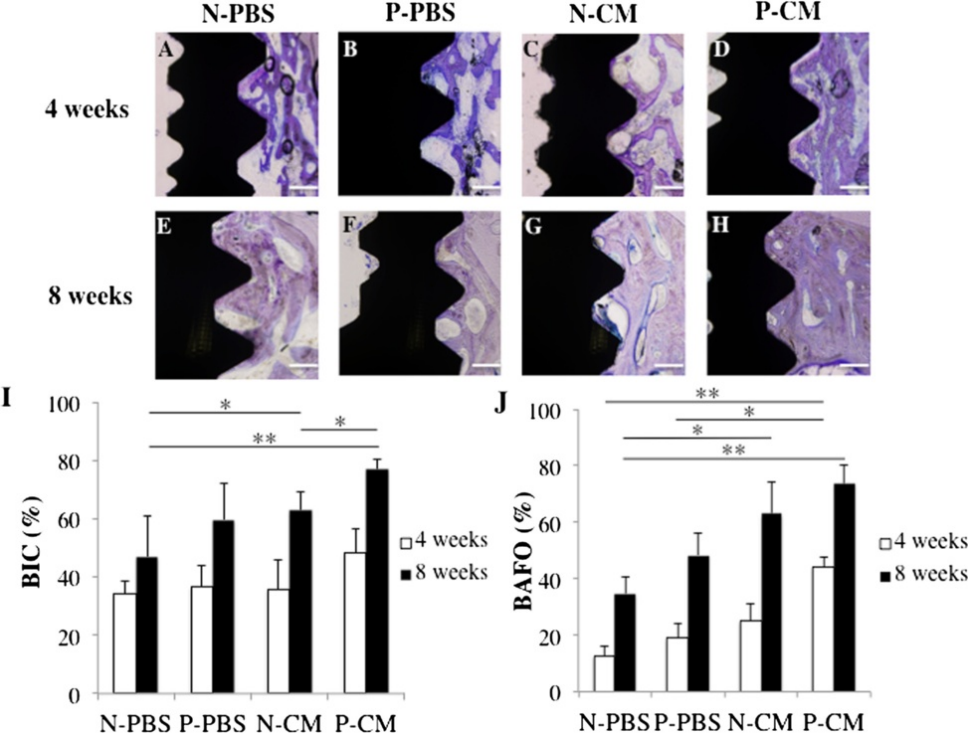
Histological analysis around the Ti implant in vivo*.* In vivo bone morphogenesis around Ti implants as observed under × 100 magnification at 4 weeks after implantation (**a**–**d**) and 8 weeks after implantation (**e**–**h**). (**a**, **e**) N-PBS implants, (**b**, **f**) P-PBS implants, (**c**, **g**) N-CM implants, (**d**, **h**) P-CM implants. Bar indicates 100 μm. Average histomorphometric values of bone implant contact (BIC) (**i**) and bone area fraction occupancy (BAFO) (**j**). Data are presented as mean ± SD (n = 3) for panels (**i**) and (**j**). *P < 0.05, **P < 0.01. *N-CM* plasma-untreated Ti on which SHED-CM was immobilized, *N-PBS* plasma-untreated Ti on which PBS was immobilized, *P-CM* Plasma-treated Ti on which SHED-CM was immobilized, *P-PBS* Plasma-treated Ti on which PBS was immobilized

Histological analysis performed 4 weeks after implantation showed continuous newly formed bone in P-CM (Fig. 7d). In other groups, bone formation was sparsely distributed around the implant fixture (Fig. 7a–c). Eight weeks post-implantation, we found more continuous newly formed bone in N-CM and P-CM groups than in N-PBS and P-PBS groups (Fig. 7e–h).

BIC and BAFO were measured in trabecular bone for quantitative histological analysis. No significant differences in BIC were observed between experimental groups at 4 weeks. However, 8 weeks after implantation, BIC was significantly higher in N-CM and P-CM groups than in N-PBS and P-PBS groups. Further, BIC was higher in P-CM than in N-CM (Fig. 7i). After 4 weeks of implantation, BAFO was significantly higher in P-CM and N-CM than in N-PBS or P-PBS groups. Further, at 8 weeks post-implantation, BAFO was significantly higher in P-CM or N-CM than in N-PBS (Fig. 7j).

## Discussion

A variety of biomolecules, including peptides, ECM proteins, and growth factors, have been used for implant surface modification [43]. Previous reports have shown that these biomolecules promote osseointegration and bone formation around the Ti implant. CM was therefore used for tissue regeneration as CM contains various biomolecules [27]. We previously showed that the CM biomolecules derived from BMSCs immobilized on the Ti surface and promoted osseointegration [31]. In this study, SHED-CM was immobilized on the surface of Ti because SHED had a higher ability for bone regeneration than BMSCs [33, 34]. APP treatment was used in this study in order to immobilize soluble proteins like CM onto Ti. Thus, we attempted to promote both bone formation and osseointegration by using a combination of SHED-CM and APP treatment.

The SEM images of Ti implant “Brånemark MkIII TiUnite®” showed a roughness of several micrometers in thickness (Fig. 1a, e). Further, the material was porous, and had an oxide film containing phosphorus on the surface that facilitated osseointegration [44, 45] . SEM images of the implant surface after APP processing showed no significant changes (Fig. 1b, f). The result showed that APP treatment did not alter the surface structure of the Ti implant. The SEM images and EDX analysis suggested that these deposits formed calcium phosphate (Ca-P) because the Ca and P signals were overlapping (Fig. 1c, d, g, h; Fig. 2a, b). In a previous study, Ca-P was deposited for 1 hour in a pH 7.4 electrolyte solution containing calcium ion and phosphate ion, corresponded to the presence of bodily fluids on the Ti discs [46]. The phosphorous solution contained a number of compounds including calcium chloride, components of the basic medium in the SHED-CM, inorganic salts such as monosodium phosphate and sodium bicarbonate, the oxide film, and CO_2_. Calcium and phosphate deposition during the 24 hours incubation of Ti in SHED-CM, at 37 °C and pH 7.45, led to precipitation of inorganic compounds. On the other hand, deposits on the Ti implant treated with DMEM were also observed [32]. However, the morphology and the amount of deposits treated with DMEM were different from that for the SHED-CM group. This finding suggests that the mixture of inorganic and organic components in SHED-CM form different morphologies.

The proteins derived from SHED-CM on Ti particles were detected in the only guanidine extract by using SDS-PAGE, EDX, and SEM analyses (Fig. 3a, b). On the other hand, Ca-P components were not detected in the guanidine and EDTA extract (Fig. 3c, d). These results suggested that the proteins were immobilized directly onto the Ti surface. Further, protein immobilization did not require calcium involvement. In previous reports, proteins containing glycosaminoglycan were adsorbed through calcium [47]. However, calcium-binding proteins, such as osteocalcin and bone sialoprotein, were not detected in the EDTA extract in this study.

The results of LC/MS/MS showed that ECM proteins were the main components of the CM derived from SHED (Tables 1, 2 and 3). However, some of the cytokine and growth factors in SHED-CM were different from the CM derived from BMSCs [32]. Immobilization of type I collagen on Ti has been reported as primarily involving van der Waals forces and hydrogen bonds between the proline of type I collagen molecules and Ti [48]. These reports agree with our results.

The results of the silver staining and Bradford protein assay showed improved immobilization of proteins on the Ti surface following APP pretreatment. In this study, only Ti powder was used for analysis of CM component adsorption. There were no remarkable differences in the amount of protein attached to different types of Ti materials. Further, this attachment depends more on the surface topology or chemical properties, such as hydrophilicity treatment, than different types of Ti material [17, 49, 50]. Therefore, we consider the Ti powder to be an appropriate carrier for the protein attachment assay. Within 4 weeks of its initial fabrication, Ti goes through an aging process where the hydrophilic surface of Ti becomes hydrophobic [51]. This aging process is a result of organic matter adsorption on the implant surface similar to hydrocarbon adsorption. This aging process leads to negative effects on osteoblast proliferation and differentiation because hydrocarbon disturbs protein immobilization [17]. Increased protein adsorption on Ti decreased hydrocarbon attachment on the Ti surface [51]. The APP pretreatment removed this hydrocarbon, preventing aging. This is similar to the method that UV and hydrothermal treatments utilize to prevent aging [52]. In addition, it was found that the adsorption of fibronectin onto the Ti surface increased under hydrophilic conditions [53, 54]. These results suggested that the elimination of organic matter from the Ti surface improved hydrophilicity and increased the immobilization of proteins derived from SHED-CM on the Ti surface. In a previous study, we analyzed the localization of rat BMSC-CM immobilized on Ti implants after implantation by in vivo imaging [32]. Fluorescence signals were detected in the BMSC-CM-treated groups at 28 days post-implantation, and confirmed the localization of BMSC-CM around Ti implants. Overall, fluorescence signals gradually decreased in a time-dependent manner in the BMSC-CM-treated groups. The existence of CM was confirmed on Ti implants on day 28. These results show that the proteins derived from SHED-CM on the Ti implants function on neighboring cells for at least 28 days.

The number of cBMSCs attached to Ti discs increased after 24 hours in the P-CM groups (Fig. 5). This was likely due to the deposition of fibronectin and type I collagen on the Ti surface in the P-CM groups as both type I collagen and laminin-5 promote adhesion of hMSCs [55]. Further, fibronectin is also known to promote cell adhesion. The number of osteogenic cells immobilized onto Ti increased when the surface was treated with fibronectin [23]. On the other hand, the number of cBMSCs attached to P-CM was not significantly higher than the number of cBMSCs attached to N-CM. This result meant that the quantity of protein immobilized on the Ti disc did not impact cell attachment. SEM and EDX analysis suggested that the ECM area was partially covered by Ca-P. This meant that the ECM area available for cell attachment was no different from the ECM area available in the N-CM groups. Further investigation is warranted to utilize these ECM components more effectively. In this study, APP treatment did not significantly improve cell attachment in both the PBS and SHED-CM groups. It has previously been shown that hydrophilicity treatment improves fibronectin adsorption from serum, thereby promoting cell attachment [17]. We considered the possibility that components of PBS (i.e., primarily Na^+^ or Cl^−^) bound to the substrate and inhibited fibronectin adsorption in the P-PBS groups. On the other hand, the surface area of Ti discs with immobilized SHED-CM was similar in the P-CM and N-CM groups; however, the amount of protein from SHED-CM on Ti discs increased. The amount of protein from SHED-CM increased the in P-CM groups, although the surface area of Ti discs with immobilized SHED-CM was similar for N-CM and P-CM groups. These observations suggest that the immobilized protein was stratified on Ti discs. Taken together, these results show that there was no difference in surface area in those groups exhibiting cell attachment (N-CM and P-CM groups); however, the amount of SHED-CM immobilized on the Ti surface was greater in the P-CM groups.

The results of the in vivo study demonstrate that the Ti implants in the P-CM groups promotes bone morphogenesis around the implant surface at 4 and 8 weeks after implantation. Ti implants are placed mainly in contact with trabecular bone; knowledge of the mechanical properties of the trabecular bone may enhance our fundamental understanding of the cause of the higher failure rates in poor quality bone [56]. Therefore, we chose trabecular bone to evaluate bone regeneration around Ti implants in this study. These results suggest that the Ti implant treated with APP and SHED-CM had higher osteoconducivity than other implant groups. This was likely due to the effect of Ca-P components and the ECM proteins such as type I collagen, fibronectin, and decorin that are immobilized on the Ti implant. Ca-P has been used for implant surface modification because of its strong resemblance to the inorganic phase of the bone matrix. Ca-P has been reported to improve osteogenic cell attachment [57], enhance osteoblast differentiation [58], and stimulate intracellular signaling pathways of osteoblast as well as calcium sensing receptors (CaSRs) [59, 60]. Additionally, Ca-P is used for implant surface modification with type I collagen, fibronectin, other ECM proteins, and RGD peptide. The collagen works as a scaffold for MSCs, and influences adhesion, migration, and differentiation of these MSCs [56]. Fibronectin and RGD peptide increase the number of adsorbed MSCs and assist in early-stage cell differentiation [23, 28]. A recent study showed that implants coated with HA and type I collagen display greater ability to stimulate new bone formation than those treated with HA or type I collagen alone [61]. In addition, histological analysis showed that BAFO was already higher in the P-CM than the N-PBS and P-PBS groups from 4 weeks after implantation. These results showed greater bone morphogenesis at places distant from the implant interface. We could not demonstrate that BIC and BAFO were significantly higher in P-CM groups than in P-PBS groups at 8 weeks post-implantation. From the standpoint of animal protection we were not able to show statistical significance without increasing the number of experiments. However, BIC and BAFO tended to show increased P-CM groups relative to P-PBS groups at 8 weeks post-implantation, and the micro-CT results and histological images support this tendency. In cell attachment assays, it was not revealed whether APP treatment significantly improved cell attachment between N-CM and P-CM groups. It may be suggested that the surface area of immobilized SHED-CM is not significantly higher in P-PBS groups than in N-CM groups; however, there was an increased amount of protein from SHED-CM on Ti discs. A previous report showed that the Ti implant treated with DMEM only had lower osteogenesis compared to BMSC-CM, but had higher osteogenesis than the control [32].

It is difficult to conclude whether inorganic or organic molecules are the main contributor to osteogenesis stimulation around the Ti implant. This is the first report that focused on inorganic molecules from SHED-CM to regenerate bone. In a previous report, we compared the osteogenic potential of BMSCs and SHED [36]. We found that osteogenic gene expression was significantly elevated in SHED compared to BMSCs. Further, we found that the BMP signaling pathway was important for bone formation [36]. Further studies are required to determine the primary protein needed to achieve high osteoconductivity in SHED-CM. In addition, studies are needed to compare bone morphogenesis between SHED-CM and BMSC-CM. These results suggest the potential for a new type of implant that has high osteoconductive ability. In the future, these highly osteoconductive implants could potentially be used to promote osteogenesis for advanced alveolar bone loss without an extra operation on bone regeneration using bone prosthetic materials.

## Conclusions

Our results showed that APP treatment promoted the immobilization of Ca-P components and ECM proteins derived from SHED-CM onto TiO_2_. Therefore, Ti implants treated with APP and SHED-CM promoted bone morphogenesis not only around the implant interface, but also at distant locations from the implant surface during the early stages of osseointegration. Our results suggest that immobilizing SHED-CM by using APP treatment may be used as an effective application to facilitate bone regeneration around dental implants.

## Abbreviations

APP: Atmospheric pressure plasma
BAFO: Bone area fraction occupancy
BIC: Bone-implant contact
BMSC: Bone marrow stromal cell
Ca-P: Calcium phosphate
CBB: Coomassie Brilliant Blue
cBMSCs: Canine bone marrow stromal cells
CM: Conditioned medium
DMEM: Dulbecco’s modified Eagle’s medium
ECM: Extracellular matrix
EDX: Energy dispersive X-ray spectroscopy
FBS: Fetal bovine serum
LC/MS/MS: Liquid chromatography-ion trap mass spectrometry
MSC: Mesenchymal stem cell
MSC-CM: Mesenchymal stem cell-conditioned medium
N-CM: Plasma-untreated Ti on which SHED-CM was immobilized
N-PBS: Plasma-untreated Ti on which PBS was immobilized
PBS: Phosphate-buffered saline
P-CM: Plasma-treated Ti on which SHED-CM was immobilized
P-PBS: Plasma-treated Ti on which PBS was immobilized
SEM: Scanning electron microscopy
SEM-EDX: Scanning electron microscopy combined with energy dispersive X-ray spectroscopy
SHED: Stem cells from human exfoliated deciduous teeth
SHED-CM: Stem cells from human exfoliated deciduous teeth-conditioned medium
Ti: Titanium
